# Efficacy of Initial vs. Delayed Photodynamic Therapy in Combination With Conbercept for Polypoidal Choroidal Vasculopathy

**DOI:** 10.3389/fmed.2021.791935

**Published:** 2022-02-09

**Authors:** Zuhua Sun, Yuanyuan Gong, Yating Yang, Ying Huang, Suqin Yu, Junqing Pei, Bing Lin, Rong Zhou, Yingzi Li, Yumin Li, Junyan Zhang, Xiaoling Liu

**Affiliations:** ^1^School of Ophthalmology & Optometry and Eye Hospital, Wenzhou Medical University, Wenzhou, China; ^2^Department of Ophthalmology, Shanghai General Hospital, Shanghai Jiao Tong University School of Medicine, Shanghai, China; ^3^Department of Ophthalmology, Sir Run Run Shaw Hospital, Medical College of Zhejiang University, Hangzhou, China; ^4^Bothwin Clinical Study Consultant, Shanghai, China

**Keywords:** polypoidal choroidal vasculopathy, photodynamic therapy, conbercept, non-inferiority, efficacy

## Abstract

**Purpose:**

To compare the efficacy of initial vs. delayed photodynamic therapy (PDT) in combination with intravitreal injection of conbercept (IVC) for polypoidal choroidal vasculopathy (PCV).

**Design:**

Multicenter, randomized, non-inferiority clinical trial.

**Subjects:**

Naïve PCV patients.

**Methods:**

Patients were randomized 1:1 into two groups: initial PDT with IVC and delayed PDT with IVC. At baseline, patients in the initial combination group were treated with PDT and IVC within 1 week, while patients in the delayed combination group were treated with IVC alone. PDT and IVC was given PRN during the follow-up in each group.

**Main Outcome Measures:**

Non-inferiority of delayed PDT with IVC to initial PDT with IVC for mean change in best-corrected visual acuity from baseline to month 12 (95% CI of the difference entirely above −5 letters).

**Results:**

Eighty-six patients were enrolled, with 43 in each group. At month 12, the change of BCVA in initial combination group was equivalent to that in the delayed combination group, with gains of 6.42 ± 1.89 and 7.49 ± 2.14 (mean ± standard error) letters, respectively [delayed group minus initial group: 1.07 letters; 95% confidence interval (CI): −4.62 to 6.76; *P*_*non*−*inferiority*_ = 0.0198]. The rates of complete polyp regression were 66.67 and 45.83% in the initial and delayed combination groups, respectively. The difference was not statistically significant (*P* = 0.386). The mean reductions of CRT were 204.77 ± 28.79 and 84.14 ± 30.62 μm in each group respectively. The difference was statistically significant (*P* = 0.005). In addition, the mean injection numbers were 3.47 ± 2.39 and 4.91 ± 2.65 in each group respectively. The differences were statistically significant (*P* = 0.010).

**Conclusions:**

There was effective in both groups in patients with PCV. The initial combination group showed a more efficient decrease in CRT and polyp regression, along with fewer injections. However, the delayed combination group was non-inferior compared with the initial combination group in terms of the improvement of BCVA.

**Trial Registration:**

https://ClinicalTrials.gov, Identifier: NCT02821520.

## Introduction

Age-related macular degeneration (AMD) is the leading cause of blindness worldwide and is expected to affect 170 million people by 2040, including 110 million people in Asia alone (1–3). Polypoidal choroidal vasculopathy (PCV) has been considered as a subtype of neovascular AMD, in which type I neovascularization is related to the abnormal branching vascular network (BVN) and the expansion of aneurysms referred to as polyps occurs (4). PCV is more prevalent among Asian patients than among Caucasians. Nearly half of Chinese patients diagnosed with neovascular AMD actually have PCV, while among Caucasian patients, the proportion is only about 10% (5). Submacular hemorrhage and serous exudation, as well as multiple and recurrent detachment of retinal pigment epithelium (RPE) and neurosensory retina, are common features in PCV patients.

Recently, the combination of anti-vascular endothelial growth factor (VEGF) and photodynamic therapy (PDT) has been recommended as a feasible regimen for PCV to achieve a synergistic therapeutic effect. Their benefits to vision have been confirmed regardless of ethnicity or disease subtype (6–11). PDT can reduce the thickness of the macula in the early stage of treatment, although it has been reported to result in sight threatening complications such as subretinal, vitreous and suprachoroidal hemorrhage, as well as tears and rips of the RPE. While a recently study showed none of the patients in either the standard-fluence PDT group nor the reduced-fluence PDT group suffered from any major complication, and the effect in each group was comparable (12). On the other hand, the intravitreal injection of anti-VEGF agents can significantly improve vision in the long term. Previous high-quality clinical trials, including the EVEREST I&II, LAPTOP, FUJISAN, and PLANET studies, found that anti-VEGF therapy could induce a desirable improvement in BCVA (7–11). However, their effects in achieving polyp regression and reducing BVN are not satisfactory. Consequently, combining these two methods may make up for the shortcomings of monotherapy and improve the overall efficacy for patients with PCV (7, 9, 11, 13, 14). This could have the tremendous clinical benefit of achieving a higher rate of polyp regression.

Conbercept is a novel anti-VEGF agent made in China. It is a soluble recombinant VEGF receptor that competitively binds to all subtypes of VEGF-A, VEGF-B, and placental growth factor (PIGF). Conbercept is endowed with a stronger affinity for VEGF-A than other anti-VEGF agents and can effectively treat exudative AMD (15, 16); its therapeutic efficacy has been shown to vary among PCV patients. However, previous studies were limited to a short follow-up period (17), a single-center setting, and an insufficient sample size. Besides, the optimal paradigm of PDT combined with anti-VEGF therapy was not extensively assessed, and whether PDT should be administered at the beginning of treatment or during follow-up of anti-VEGF therapy has not been determined.

Therefore, we designed and conducted a 12-month prospective, multicenter, randomized, non-inferiority clinical trial to evaluate the efficacy of initial vs. delayed PDT in combination with intravitreal injection of conbercept (IVC) for PCV patients. Some patients can obtain good effect after only one or two injections of anti VEGF therapy, especially in combination with PDT in the real-world. So, we assessed the efficacy of a dosing pattern of only one injection of conbercept and then PRN in this study.

## Subjects and Methods

This study was designed as a prospective, multicenter, randomized, non-inferiority, 12-month clinical trial comparing the efficacy of initial vs. delayed PDT in combination with IVC for PCV patients. The study was registered with ClinicalTrials.gov (identifier no. NCT02821520). Patients were recruited from the School of Ophthalmology & Optometry and Eye Hospital, Wenzhou Medical University, Shanghai General Hospital, Shanghai Jiao Tong University School of Medicine, and Sir Run Run Shaw Hospital, Medical College of Zhejiang University. The study protocol was approved by the ethics committee of each of the hospitals mentioned above. The study was carried out in compliance with the Declaration of Helsinki and the International Conference on Harmonization Guidelines for Good Clinical Practice. All patients in this trial provided written informed consent prior to any treatment. The data presented here were collected between January 2017 and May 2019.

### Participants

Patients aged ≥40 years old of either sex with naive symptomatic PCV were eligible for enrollment in this study if they had active polyps with or without an abnormal vascular network on indocyanine green angiography (ICGA) (Spectralis; Heidelberg Engineering, Heidelberg, Germany) and best-corrected visual acuity (BCVA) of 34 to 79 Early Treatment Diabetic Retinopathy Study (ETDRS) letters (Snellen Equivalent 20/200 to 20/25). Symptomatic active PCV was defined as blurred vision caused by hemorrhage or intraretinal fluid or subretinal fluid involved the fovea. The polypoidal lesions or BVN should located within the vascular arches. Patients with refractive medium opacity or small pupil that could influence the fundus examination were excluded. The women were required to be using effective contraception, be post-menopausal for at least 6 months prior to trial entry, or be surgically sterile. Patients had to have the ability to provide written informed consent and to return for all study visits. Only one eye from each patient was included as the study eye. If both of a subject's eyes meet the inclusion criteria, the eye with poor vision was selected as the study eye.

Exclusion criteria included any of the following conditions found in the study eye: active inflammation or infection; uncontrolled intraocular pressure (>25 mmHg); an ocular condition that may impact vision and confound the study outcomes (e.g., vitreomacular traction, epiretinal membrane with BCVA impact, ocular inflammation, retinal vascular diseases such as diabetic retinopathy or diabetic macular edema); the presence of central macular scarring or atrophy indicating irreversible BCVA loss; prior treatment of the study eye with anti-VEGF therapy or systemic use of anti-VEGF agents within 3 months prior to study entry; previous vitrectomy, macular laser treatment, PDT, or intraocular steroids; allergy to fluorescein, ICG, iodine, shellfish; and pregnant or breastfeeding women.

Eighty-six patients were enrolled and randomized 1:1 into the initial and delayed PDT in combination with conbercept groups, in accordance with a predetermined randomization scheme provided by a designated, blinded statistician. A secure, computer-generated randomization schedule was maintained in concealed envelopes by a study-group member who did not participate in enrollment. The concealed envelopes were revealed by treatment physicians only after eligibility for enrollment had been confirmed and before the treatment was given to the patients.

### Treatments

In the initial combination group, PDT (intravenous injection of verteporfin 6 mg/m^2^ and laser irradiation at 689-nm wavelengths and 600 mW/cm^2^ irradiance for 83 s) was administered within 1 week after the intravitreal injection of conbercept (0.5 mg /0.05 ml, Chengdu Kanghong Biotechnologies Co. Ltd, China). The PDT targets were whole lesions that including polypoidal lesions and BVN seen on ICGA. From baseline to month 11, participants were followed up monthly and received PRN IVC. From months 3 to 11, PDT was performed if they met the rescue treatment criteria; the intervals of PDT had to be no <3 months. The final follow-up was performed at month 12.

In the delayed combination group, IVC was administered first, without the intervention of PDT. Then, the follow-up and treatment regimen were performed in accordance with those in the initial combination group. PDT was performed only if the patients met the rescue treatment criteria from the 3rd month; the intervals of PDT should be no <3 months. It should be noted that the loading dose of IVC was set with only one injection in each group.

Rescue IVC treatment criteria included new or persistent subretinal/inner fluid detected by OCT; CRT increase≥50 μm compared with the last visit; BCVA decrease ≥ 5 ETDRS letters compared with the last visit; or active leakage of polypoidal lesions detected by ICGA. ICGA was performed at baseline, month 3 and month 12. During month 3 to month 11, ICGA was repeated when subretinal or inner fluid didn't decrease or worse compared with baseline. Rescue PDT treatment criteria included an increase of central retinal thickness (CRT) by ≥50 μm compared with that at baseline; new or enlarged polyps, or BVN detected by ICGA. First of all, the investigator should believe PDT might be beneficial (9, 10). If the patient's disease progression met both IVC treatment and PDT, IVC and PDT was administered within 1 week.

**Figure 1:**
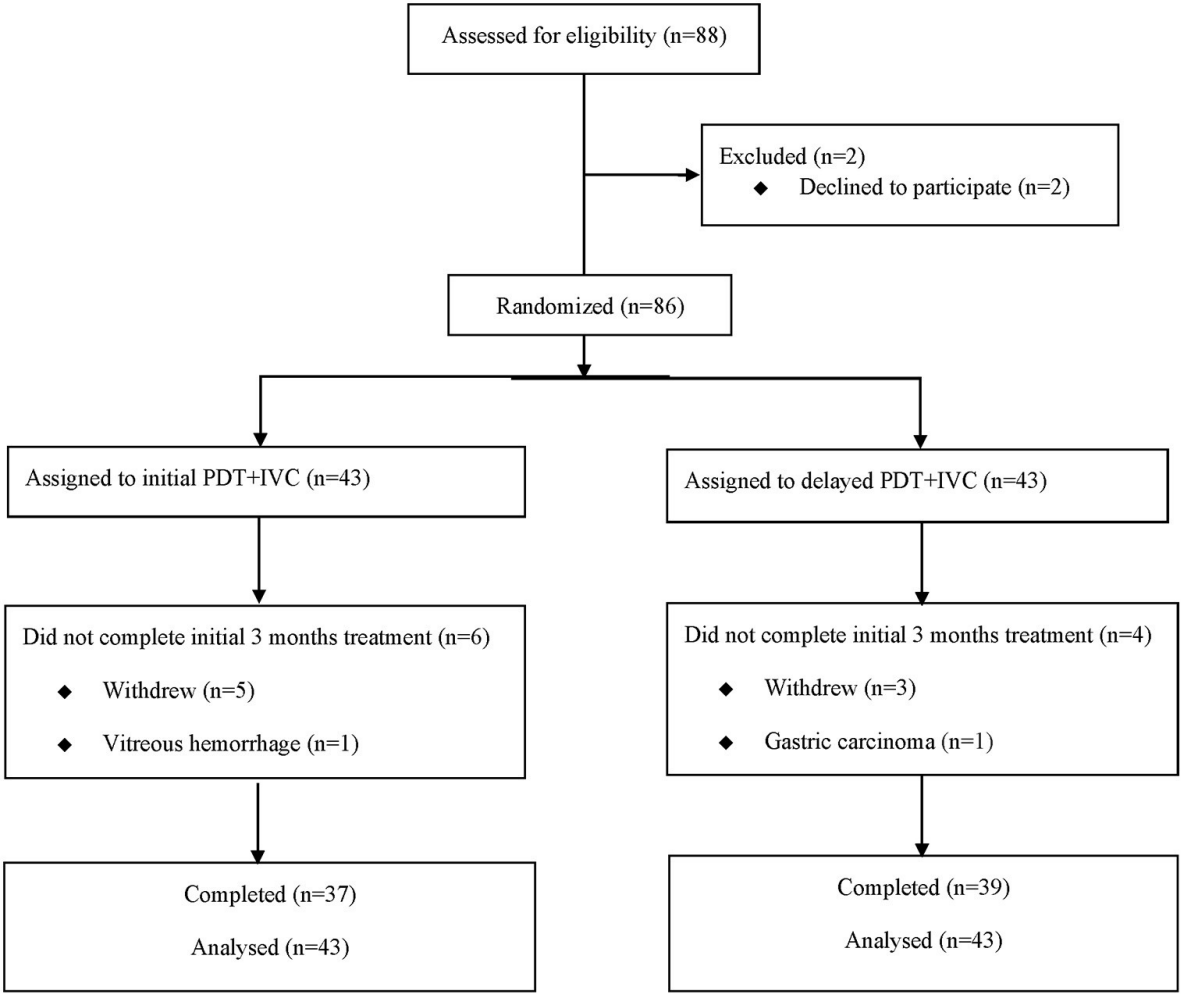
Consolidated Standards of Reporting Trials (CONSORT) diagram showing progress of patients through the study. PDT, photodynamic therapy; IVC, intravitreal injection of conbercept.

Prior to the treatment, all patients underwent ophthalmic examinations, including BCVA, anterior segment examination, dilated fundus examination, fundus photography (FP) (CR-1 Mark II; Canon, Japan), CRT measured by spectral-domain optical coherence tomography (SD-OCT) (Spectralis; Heidelberg Engineering, Heidelberg, Germany), fundus fluorescein angiography, and ICGA (Spectralis; Heidelberg Engineering, Heidelberg, Germany). Patients were followed monthly until 12 months after the treatment, with the reassessment of BCVA, fundus, and OCT. A flowchart of the follow-up is shown in Figure 1.

### Outcome Measurements

The primary efficacy outcome was the change in BCVA from baseline to month 12, in addition to the proportion of patients who had complete polyp regression at month 12, and changes in BCVA and CRT from baseline to months 3 and 12. The secondary outcomes included the proportion of patients who gained **≥**5 ETDRS letters in BCVA from baseline to month 12, as well as those who accepted retreatment through the 12-month treatment. The numbers of PDT and IVC in the two groups were also evaluated. Safety assessments included endophthalmitis, vitreous hemorrhage, and any other ocular or systemic events during the 12-month follow-up.

BCVA was assessed following the ETDRS protocol by a certified optometrist. CRT was assessed using SD-OCT by qualified masked technicians. The OCT examination included 25 sections, each of which comprised nine averaged scans and were obtained in an area of a 6 × 6 mm square centered on the fovea.

### Statistical Analysis

This study was designed as a non-inferiority trial comparing the two groups. The subjects were included in the two groups at a ratio of 1:1. It was assumed that patients in the initial combination group could increase by 9.5 ± 3.2 letters at the end of this study, while patients in the delayed combination group could show an improvement of 6.5 ± 3.2 letters. For the primary outcome, the non-inferiority limit for the difference between the two groups in the mean change in BCVA at month 12 was five letters (18). With power of 0.85 and α of 0.025 for a one-sided test, 37 patients in each group were needed. Considering a rate of loss to follow-up of 10%, recruitment of 43 patients in each group was planned.

A one-tailed statistical test for non-inferiority between the two groups was performed. The primary analysis followed the intention-to-treat (ITT) principle. Missing data were imputed using the last observation carried forward (LOCF) method. Statistical analysis of the data between baseline and follow-up in each group was performed. Means ± SD were reported. Statistical testing was conducted at a significance level of 0.025 (one-sided). Mean values of continuous variables were compared between groups using independent *t*-tests. Changes in BCVA, CRT, and central choroidal thickness of both groups from baseline to follow-up were compared using paired *t*-tests and the Wilcoxon rank-sum (Mann-Whitney) test. A chi-squared test was used to compare categorical data between the two groups.

The safety analysis was performed for all of the patients who received at least one administration of IVC or PDT and had a safety assessment followed the ITT principle. All of the adverse events were compared between the two groups using the chi-squared test or Fisher's exact method.

## Results

### Baseline Characteristics

**Figure 2:**
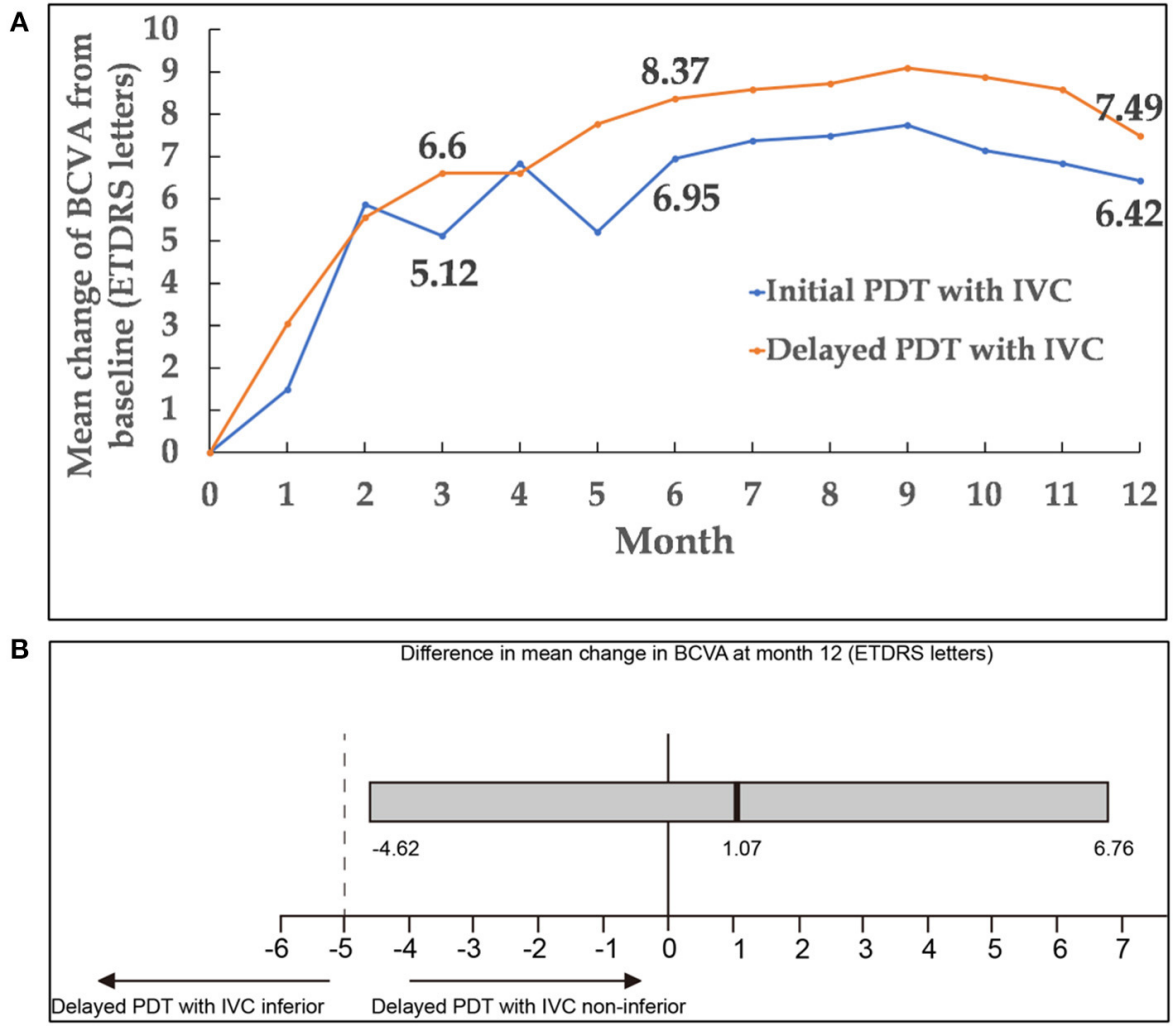
**(A)** Mean change of BCVA from baseline to month 12. **(B)** Differences in BCVA change from baseline to month 12 between the two groups. The black vertical lines indicated the mean difference between the two groups, and the gray bar was the 95.0% CI. CI within −5 and +5 letters (dashed vertical lines) indicated that the two groups were equivalent. A lower limit of the 95.0% CI with a value above −5 showed that delayed PDT combined with IVC was non-inferior compared with initial PDT combined with IVC. BCVA, best-corrected visual acuity; CI, confidence interval; PDT, photodynamic therapy; IVC, intravitreal injection of conbercept.

The study sample consisted of 86 participants (Figure 2), who were randomized into the initial combination group (*n* = 43) and the delayed combination group (*n* = 43). Fifty nine patients came from School of Ophthalmology & Optometry and Eye Hospital, Wenzhou Medical University, twenty five patients came from Shanghai General Hospital, Shanghai Jiao Tong University School of Medicine, and two patients came from Sir Run Run Shaw Hospital, Medical College of Zhejiang University. One patient in each group withdrew their informed consent before treatment. There were no significant differences concerning the baseline characteristics and ocular examinations, including age, duration of disease, BCVA, and CRT, between the two groups, except for in terms of sex (Table 1).

**Table 1:** Baseline demographics and ocular disease characteristics (randomized set).

	**Initial PDT with IVC**	**Delayed PDT with IVC**	***P* value**
Age (years) Mean ± SD	65.1 ± 8.0	64.7 ± 7.1	0.787
Gender			
Male n (%)	30 (69.8)	38 (88.4)	0.034
Female n (%)	13 (30.2)	5 (11.6)	
Duration (months) Mean ± SD	11.3 ± 25.2	8.1 ± 11.1	0.456
Baseline BCVA (ETDRS letters) Mean ± SD	58.9 ± 15.9	57.0 ± 11.7	0.533
Baseline CRT (μm) Mean ± SD	500.8 ± 205.3	440.7 ± 185.5	0.158

*PDT, photodynamic therapy; IVC, intravitreal injection of conbercept; BCVA, best corrected visual acuity; ETDRS, Early Treatment Diabetic Retinopathy Study; CRT, central retinal thickness; SD, standard deviation*.

At month 3, 10 patients dropped out of the study, either because of moving elsewhere, poor visual improvement, or stable visual acuity. Among these 10 patients, six were in the initial combination group and four were in the delayed combination group. At month 12, about 35% of patients dropped out of the study.

### Efficacy Endpoints

#### Primary Endpoint

**Figure 3:**
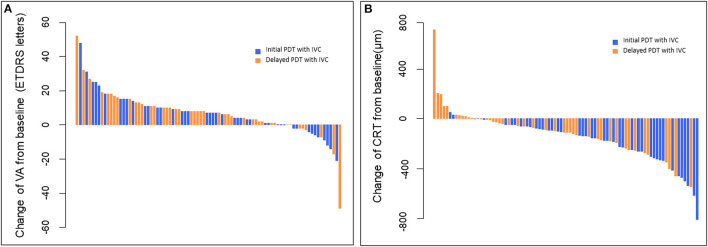
**(A)** Waterfall plots of BCVA changes from baseline to month 12 for individual patients. These plots showed that all the BCVA scores improved except 10 patients in initial PDT with IVC and 6 patients in delayed PDT with IVC. **(B)** Waterfall plots of CRT changes from baseline to month 12 for individual patients. These plots showed that all the CRT thicknesses decreased from baseline except two patients in initial PDT with IVC and 11 patients in delayed PDT with IVC. BCVA, best-corrected visual acuity; CRT, central retinal thickness; PDT, photodynamic therapy; IVC, intravitreal injection of conbercept.

Remarkable improvements in BCVA were noted during the first 3 months of treatment; thereafter, BCVA remained stable through 12 months in both groups. At month 12, the change of BCVA in initial combination group was equivalent to the delayed combination group, with gains of 6.42 ± 1.89 and 7.49 ± 2.14 (mean ± standard error) ETDRS letters in BCVA, respectively [delayed group minus initial group: 1.07 letters; 95% confidence interval (CI): −4.62 to 6.76; *P*_*non*−*inferiority*_ = 0.0198]. Mean changes of BCVA from baseline up to month 12 in both groups were presented in Figures 2, 3A. It was worth noting that BCVA dramatically improved 48 letters at month 12 in one patient after only once PDT combined with IVC in initial combination group. BCVA dramatically improved 52 letters at month 12 in another patient after three continuously IVC in delayed combination group. While it was also be noted that BCVA decreased eight letters at month 8 but dramatically decreased 49 letters at month 12 in one patient who received six injections of IVC and twice PDT in the delayed combination group. Polypoidal lesions was located at the temporal of the optic nerve with subretinal fluid involving the fovea in this patient at baseline, while new serous pigment epithelial detachment involving the fovea happened at month 6 and developed into vascular pigment epithelial detachment combined with macular edema during the follow up. What's more, the patient refused treatment after month 8 during the follow up.

#### Secondary Endpoints

**Figure 4:**
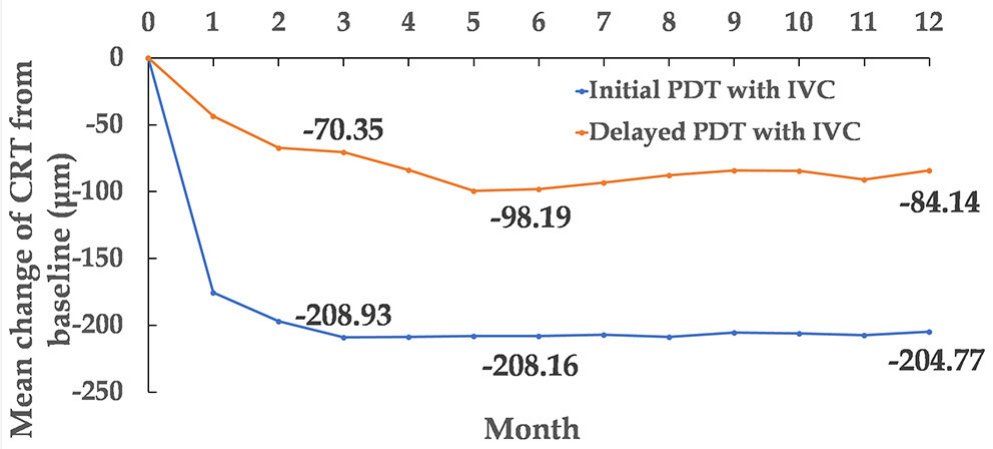
Mean change of CRT from baseline to month 12. CRT, central retinal thickness.

The CRT of the two groups was found to be significantly decreased. The decrease in CRT at month 3 was 208.93 ± 25.35 and 70.35 ± 20.93 μm (mean ± standard error) in the initial combination and delayed combination group, respectively. The difference was statistically significant between the two groups (*P* < 0.001). At month 12, CRT had decreased 204.77 ± 28.79 and 84.14 ± 30.62 μm (mean ± standard error) in the initial combination and delayed combination groups, respectively. This difference was also statistically significant (*P* = 0.005) (Figures 3B, 4).

At month 3, among patients in the initial combination group, there was a significantly higher proportion achieving complete polyp regression than in the delayed combination group (56.41 vs. 17.95%). At month 12, the proportions with complete polyp regression in the two groups were 66.67 and 45.83%, respectively.

The proportions of patients who gained ≥5 ETDRS letters from baseline to month 12 were 53.49% in the initial combination group and 65.12% in the delayed combination group. The proportions of patients who dropped ≥15 letters from baseline to month 12 were 2.33% in the initial combination group and 4.65% in the delayed combination group.

During the study period, the mean numbers of PDT were 1.09 and 0.40 in the initial combination group and the delayed combination group, respectively, which were significantly different (*P* < 0.001). Fifteen times of PDT treatments were performed eventually in 12 patients (30.77%) in the delayed PDT group. Among which, PDT treatments were performed at month 3 in six patients, and at month 4 in three patients.

Besides, the mean numbers of IVC were 3.47 in the initial combination group and 4.91 in the delayed combination group; the difference between them was also statistically significant (*P* = 0.010). Over 12 months, seven patients (18.92%) received only one injection in initial combination group, while there was none in the delayed combination group. Twelve patients (32.43%) in initial combination group and five patients (12.82%) in delayed combination group received twice injections. 76.74% of the participants in the initial combination group and 67.44% in the delayed combination group underwent IVC five times or fewer, while only six (6.98%) participants in both groups underwent 10 to 11 injections.

### Safety Profiles

Vitreous hemorrhage was the only serious ocular adverse event reported in one patient in the initial combination group (2.33%), while it did not occur in the delayed combination group. No cases of retinal hole, RPE tear, or retinal detachment occurred during the follow-up. Common ocular adverse events related to the injection procedure such as subconjunctival hemorrhage, temporary eye pain, temporary intraocular pressure increase was similar to the other researches and didn't been analyzed in this study.

Systemic serious adverse events requiring hospitalization occurred in four patients (4.65%). One patient was hospitalized because of influenza and one because of allergic dermatitis in the initial group. Meanwhile, one patient was hospitalized because of gastric carcinoma and one because of fracture in the delayed group. There was no significant difference in the risks of ocular or systemic adverse events between the two groups.

## Discussion

This prospective, multicenter non-inferiority study demonstrated that both initial and delayed PDT combined with IVC could induce favorable visual outcomes in patients with PCV. The delayed combination group showed non-inferiority in the improvement of BCVA compared to the initial combination group. Approximately half of the patients gained five letters or more from baseline to month 12.

Hemorrhage and exudation of PCV lesions could affect central vision, which is prone to further damage, eventually leading to irreversible vision loss. No consensus has yet been reached on the standard treatment strategy for PCV; PDT and anti-VEGF therapy are both considered as effective methods (19, 20). Several studies have reported that PDT can effectively seal polypoid lesions (21–23). In addition, studies have shown that PDT can upregulate VEGF expression, causing complications such as subretinal hemorrhage, RPE tear, and retinal atrophy. Matsuoka et al. found high expression of VEGF in RPE cells and vascular endothelial cells in the eyes among patients with PCVs (24). By directly suppressing the VEGF highly expressed among PCV patients, anti-VEGF agents were confirmed to distinctly improve their vision, and decrease the exudation and the thickness of the fovea (25–28).

When PDT and anti-VEGF are administered in combination for PCV, the combination method and the number of injections of anti-VEGF vary. Most studies used PDT combined with three monthly injections. However, in our study, only one injection was combined with PDT in the initial treatment and then PRN. Although no differences in BCVA improvement and complete polyp regression rate were identified between the two groups at month 12, CRT rapidly decreased and polyps regressed at an early stage in the initial combination group at month 3, with fewer injections than in other studies. This suggested that the early combination of PDT and ICV can achieve an early response to treatment in patients with PCV.

Superiority in the rate of complete polyp regression was identified in the initial combination group at 3 months of treatment. However, no significant differences were found between the two groups in the rate of complete polyp regression at month 12. Our study showed that the mean number of IVC in the initial combination group was 3.47, which was significantly <4.91 in the delayed combination group. The initial combination group was proven to have a significantly higher complete polyp regression rate and a fewer number of treatments. It was similar to the results of EVEREST II study, in which PDT combined with ranibizumab can reach the better BCVA change (9.6 vs. 5.5 letters) and higher polypoidal lesion regression (56.6 vs. 26.7%) and fewer injections (6 VS 12) compared with ranibizumab monotherapy at month 24 (11). The Fujisan Study showing that the mean injections were 4.5 and 6.8 in initial PDT combined with ranibizumab group and later PDT combined with ranibizumab group with BCVA gain of 8.1 and 8.8 letters, respectively (9). The PLANET Study showing that the mean injections of aflibercept were 8.1 and 8.0 in aflibercept monotherapy and aflibercept /PDT groups with BCVA gain of 10.7 and 10.8 letters, respectively (10). The improvement of BCVA was fewer in each group in this study than that in other trials. The mean injection numbers were also fewer than that in other trials. One potential reason for these findings is that the treatment regimen involved one injection and then PRN, instead of three loading injections, in our study. Another potential reason is the high rate of loss to follow-up among the participants. A third potential reason is undertreatment. Some patients refused to continue IVC or PDT because of little improvement or worse of BCVA as well as the high treatment cost although they met the retreatment criteria during the follow up. Besides, conbercept was found to exert a stronger affinity for VEGF-A than other anti-VEGF drugs, which may have contributed to the superiority in the number of injections. The reduction in the number of injections was similar to that observed in other studies evaluating the efficacy of combination therapies for PCV patients (8). Initial combination therapy may thus help reduce the expense of anti-VEGF therapy and even that of overall treatment.

The safety profile of the PDT combined with IVC treatment was consistent with the previously established safety profile of PDT and anti-VEGF agents. Only one case of a serious ocular adverse event was found during the study.

A limitation of this study is that the follow-up period was relatively short, lasting only 12 months. Recurrences are relatively common in PCV, so a long-term follow-up period might add clinical value in future studies. We are also screening the participants to ensure the long-term efficacy and safety. Another limitation of this work is the high rate of loss to finish the follow-up of 12 months.

In conclusion, the present 12-month multicenter, randomized non-inferiority clinical study confirmed the efficacy of combination therapy of intravitreal conbercept and PDT for treating PCV. The delayed combination was non-inferior to the initial combination for improving BCVA. Notably, the initial combination treatment was more efficient at reducing CRT, with fewer PDT and conbercept injections, which may be more promising in the clinical intervention for PCV. Furthermore, one injection of conbercept and subsequent PRN therapy might be considered as a more efficient paradigm in the treatment of PCV.

## Data Availability Statement

The original contributions presented in the study are included in the article/supplementary material, further inquiries can be directed to the corresponding author.

## Ethics Statement

The studies involving human participants were reviewed and approved by the Ethics Committee of School of Ophthalmology & Optometry and Eye Hospital, Wenzhou Medical University; Shanghai General Hospital, Shanghai Jiao Tong University School of Medicine, and Sir Run Run Shaw Hospital, Medical College of Zhejiang University. The patients/participants provided their written informed consent to participate in this study.

## Author Contributions

XL had full access to all of the data in the study and takes responsibility for the integrity of the data and the accuracy of the data analysis and supervised the study. XL and ZS: study concept and design, drafting of the manuscript, critical revision of the manuscript for important intellectual content, and obtained funding. XL, ZS, YG, and YL: acquisition, analysis, or interpretation of data, administrative, technical, or material support. JZ: statistical analysis. All authors contributed to the article and approved the submitted version.

## Funding

Science and Technology Project of Wenzhou Science and Technology Bureau (Y20160448); Key Subject of Eye Hospital, Wenzhou Medical University (YNZD201601); IIT project of Research and Development Fund of Conbercept, funded by Beijing Bethune Charitable Foundation in 2017. The funding sources had no role in the design and conduct of the study; collection, management, analysis, and interpretation of the data; preparation, review, or approval of the manuscript; and decision to submit the manuscript for publication.

## Conflict of Interest

JZ was employed by the company Bothwin Clinical Study Consultant Inc. The remaining authors declare that the research was conducted in the absence of any commercial or financial relationships that could be construed as a potential conflict of interest.

## Publisher's Note

All claims expressed in this article are solely those of the authors and do not necessarily represent those of their affiliated organizations, or those of the publisher, the editors and the reviewers. Any product that may be evaluated in this article, or claim that may be made by its manufacturer, is not guaranteed or endorsed by the publisher.
