# Corrigendum: Efficacy of Initial vs. Delayed Photodynamic Therapy in Combination With Conbercept for Polypoidal Choroidal Vasculopathy

**DOI:** 10.3389/fmed.2022.899310

**Published:** 2022-06-23

**Authors:** Zuhua Sun, Yuanyuan Gong, Yating Yang, Ying Huang, Suqin Yu, Junqing Pei, Bing Lin, Rong Zhou, Yingzi Li, Yumin Li, Junyan Zhang, Xiaoling Liu

**Affiliations:** ^1^School of Ophthalmology & Optometry and Eye Hospital, Wenzhou Medical University, Wenzhou, China; ^2^Department of Ophthalmology, Shanghai General Hospital, Shanghai Jiao Tong University School of Medicine, Shanghai, China; ^3^Department of Ophthalmology, Sir Run Run Shaw Hospital, Medical College of Zhejiang University, Hangzhou, China; ^4^Bothwin Clinical Study Consultant, Shanghai, China

**Keywords:** polypoidal choroidal vasculopathy, photodynamic therapy, conbercept, non-inferiority, efficacy

In the published article, there was an error.

A correction has been made to **Abstract**, “*Conclusions:*”

This sentence previously stated:

“However, the initial combination group was non-inferior compared with the delayed combination group in terms of the improvement of BCVA.”

The corrected sentence appears below:

“However, the delayed combination group was non-inferior compared with the initial combination group in terms of the improvement of BCVA.”

In the original article, there was an error.

A correction has been made to **Conflict of Interest:**

This sentence previously stated:

“The authors declare that the research was conducted in the absence of any commercial or financial relationships that could be construed as a potential conflict of interest.”

The statement should read:

“JZ was employed by the company Bothwin Clinical Study Consultant Inc.

The remaining authors declare that the research was conducted in the absence of any commercial or financial relationships that could be construed as a potential conflict of interest.”

The authors apologize for these errors and state that this does not change the scientific conclusions of the article in any way. The original article has been updated.

## Publisher's Note

All claims expressed in this article are solely those of the authors and do not necessarily represent those of their affiliated organizations, or those of the publisher, the editors and the reviewers. Any product that may be evaluated in this article, or claim that may be made by its manufacturer, is not guaranteed or endorsed by the publisher.

